# MTP18 overexpression contributes to tumor growth and metastasis and associates with poor survival in hepatocellular carcinoma

**DOI:** 10.1038/s41419-018-0987-x

**Published:** 2018-09-20

**Authors:** Yu Zhang, Hui Li, Hulin Chang, Lixue Du, Jun Hai, Xilin Geng, Xiang Yan

**Affiliations:** Department of Hepatobiliary Surgery, Shaanxi Provincial People’s Hospital, Xi’an, 710068 China

## Abstract

**Background:**

Human MTP18 (mitochondrial protein 18 kDa) is a novel nuclear-encoded mitochondrial membrane protein that is involved in controlling mitochondrial fission. Our bioinformatic analysis of TCGA data revealed an aberrant overexpression of MTP18 in hepatocellular carcinoma (HCC). We analyzed its biological effects and prognostic significance in this malignancy.

**Methods:**

MTP18 expression was evaluated by qRT-PCR and western blot analysis in 20 paired tumor and peritumor tissues. Clinical impact of MTP18 overexpression was assessed in 156 patients with HCC. The effects of MTP18 knockdown or overexpression on cell growth and metastasis were determined by cell proliferation, colony formation, cell cycle, apoptosis, migration, and invasion assays. Furthermore, the underlying molecular mechanisms by which MTP18 overexpression promoted HCC cell growth and metastasis were explored.

**Results:**

MTP18 was commonly overexpressed in HCC tissues mainly due to the downregulation of miR-125b, which significantly contributed to poor prognosis of HCC patients. Functional experiments revealed that MTP18 promoted both the growth and metastasis of HCC cells by inducing the progression of cell cycle, epithelial to mesenchymal transition (EMT) and production of MMP–9, and suppressing cell apoptosis. Mechanistically, increased mitochondrial fission and subsequent ROS production was found to be involved in the promotion of growth and metastasis by MTP18 in HCC cells.

**Conclusions:**

MTP18 plays a pivotal oncogenic role in hepatocellular carcinogenesis; its overexpression may serve as a novel prognostic factor and a therapeutic target in HCC.

## Introduction

Liver cancer, primarily hepatocellular carcinoma (HCC), is now the second leading cause of cancer death worldwide^1^. The prognosis of patients with HCC continues to be poor despite advances in diagnostic techniques, and surgical and adjuvant systemic treatment^2^. Mitochondria are important bioenergetic and biosynthetic organelles critical for normal cell function and human health. Altered mitochondrial function has been considered as a hallmark for many types of cancer^3,4^, including HCC^5^. Identification of novel molecular regulators involved in the disruption of mitochondrial function may provide insights into the biological basis of cancer development. This is also important for revealing new diagnostic markers and therapeutic targets for treatment of this disease.

MTP18, also known as mitochondrial fission protein 1 (MTFP1), is a novel nuclear-encoded and mitochondrial localized protein that has been reported to contribute to mitochondrial fission^6^. Increasing lines of evidence indicate the close links between imbalanced mitochondrial fission/fusion and cancers^7,8^. Several studies have demonstrated that the expression of mitochondrial fission/fusion proteins such as DRP1, MFN1, and MFN2 is dysregulated in human cancers of breast, lung, and bladder, respectively^9–11^. In addition, a few recent studies have demonstrated that increased mitochondrial fission promotes cell survival of HCC cells^12,13^, indicating the involvement of mitochondrial fission in HCC progression. However, the expression and biological effects of MTP18, a novel regulator of mitochondrial fission, in cancer development is unknown, especially in HCC.

Our bioinformatic analysis of The Cancer Genome Atlas (TCGA) data revealed an aberrant overexpression of MTP18 in HCC, indicating that overexpression of MTP18 may play an important role in the progression of HCC. We conducted the first study on MTP18 in HCC focused on its biological effects and the underlying molecular mechanisms, and its prognostic significance in this malignancy.

## Results

### MTP18 is overexpressed in HCC cells and contributes to tumor progression and worse prognosis

**Fig. 1:**
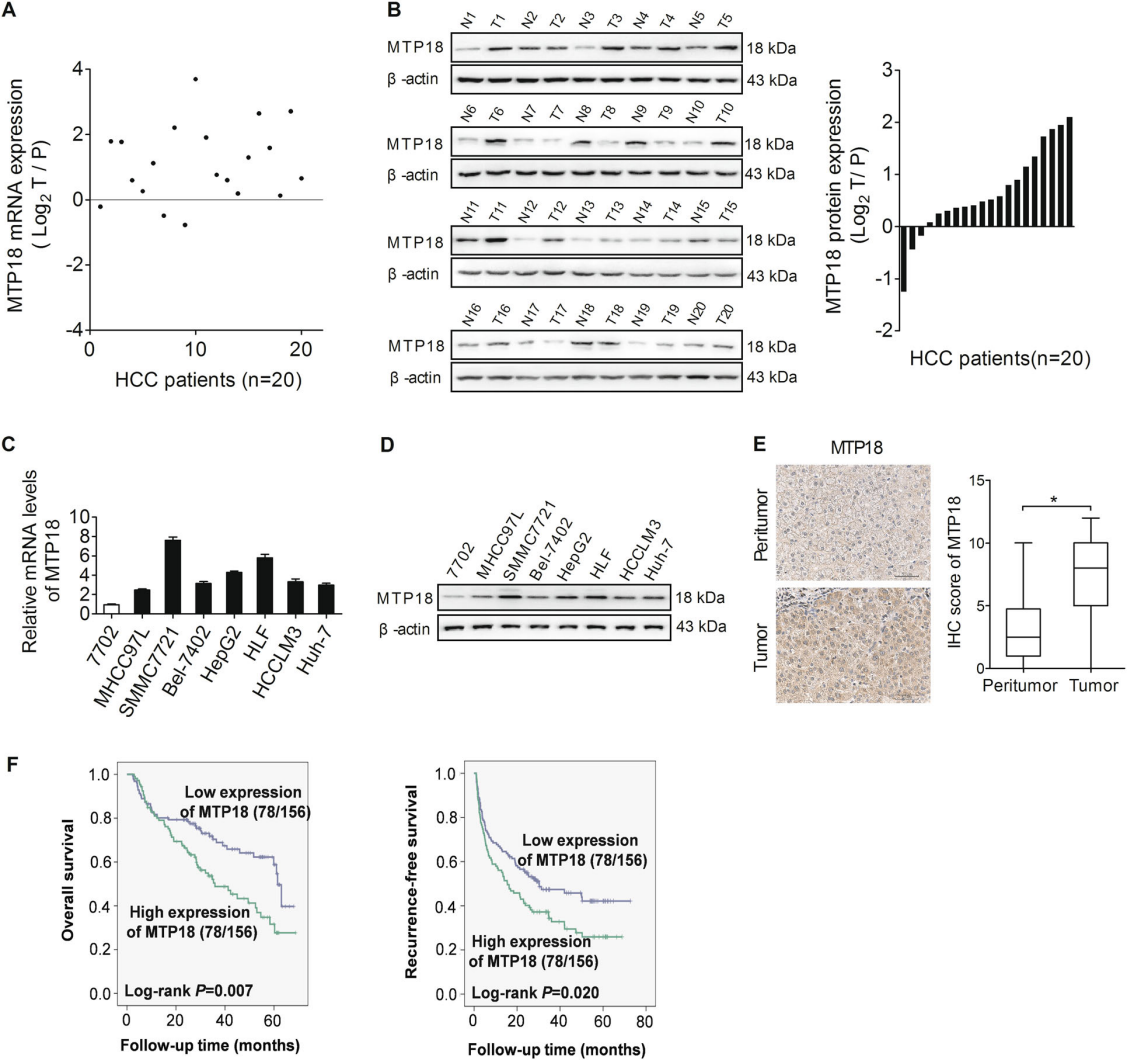
MTP18 is overexpressed in HCC cell lines and tumor tissues. **a**, **b** Quantitative real-time PCR (qRT-PCR) and western blot analyses for mRNA and protein expression levels of MTP18 in the tumor tissues and paired peritumor tissues of 20 HCC patients. (T tumor, P peritumor) Scale bars, 50 μm. The relative MTP18 expression ratio of tumor to peritumor was log2-transformed. **c**, **d** qRT-PCR and western blot analyses for mRNA and protein expression levels of MTP18 in 7 HCC cell lines (MHCC97L, SMMC7721, Bel-7402, HepG2, HLF, HCCLM3, and Huh-7). **e** Left panel: Representative immunohistochemical (IHC) staining images for MTP18 in paired tumor and peritumor tissues of HCC. Scale bar, 50 μm. Right panel: IHC staining intensity for MTP18 in 156 paired tumor tissues and peritumor tissues (*n* = 156). **f** Kaplan–Meier curves analysis for overall survival (OS) and recurrence-free survival (RFS) in tumor tissues from 156 HCC patients according to MTP18 expression levels. Data were presented as the mean ± SEM from three independent repeats, *P < 0.05

Bioinformatic analysis based on the public mRNA expression data set of TCGA showed a significant increase of MTP18 expression in HCC tumor tissues as compared to peritumor tissues (Fig. S1). To validate the results of bioinformatic analysis, we determined the expression levels of MTP18 by quantitative real-time PCR (qRT-PCR) and western blot analysis in 20 paired HCC tissues. Our results showed a significantly upregulated MTP18 in HCC tissues when compared with peritumor tissues (Fig. 1a, b). In concordance with the results from HCC tissues, the expression levels of MTP18 were significantly higher in seven HCC cell lines (HepG2, SMMC7721, MHCC97L, Bel-7402, Huh-7, HCCLM3 and HLF) when compared with normal hepatocytes (HL-7702 cells) (Fig. 1c, d).

To evaluate the clinical significance of MTP18 in HCC, MTP18 expression was evaluated by immunohistochemical staining analysis in 156 HCC patients. Representative MTP18 staining images and intensity scores (Fig. 1e) demonstrated that MTP18 is significantly higher in HCC tissues compared with adjacent non-tumor tissues, which further confirmed the results from qRT-PCR and western blot (shown in Fig. 1a, b). Correlation analysis between the expression levels of MTP18 and clinicopathological features such as age, gender, virus infection, serum alpha fetoprotein (AFP), tumor size, TNM stage and tumor differentiation showed that higher expression of MTP18 was significantly associated with higher incidence of portal vein tumor thrombosis (PVTT) and larger tumor size, while no correlation was found between the expression levels of MTP18 and other clinicopathological features (Table 1). Moreover, HCC patients with high MTP18 expression had significantly poorer overall survival (OS) and recurrence-free survival (RFS) than those with low MTP18 expression, as evidenced by Kaplan-Meier survival curves (Fig. 1f). These findings collectively indicate that MTP18 is overexpressed in HCC cells, which predicts a poor prognosis in HCC.

**Table 1:** Correlation of MTP18 expression with clinicopathologic features in patients with hepatocellular carcinoma

Variables	No. of cases (%)	MTP18 expression		*P* value
		Low	High	
All	156 (100%)	78	78	
Age				
<55	68 (43.6%)	32	36	0.628
≥55	88(56.4%)	46	42	
Gender				
Female	21 (13.5%)	13	8	0.348
Male	135 (86.5%)	65	70	
HBsAg				
Negative	15 (9.6%)	9	6	0.588
Positive	141 (90.4%)	69	72	
AFP (µg/ml)				
<200	85 (54.5%)	41	44	0.748
≥200	71 (44.5%)	37	34	
Maximum diameter of lesion				
< 5	127 (81.4%)	69	58	**0.038**
≥5	29 (18.6%)	9	20	
PVTT				
No	135 (86.5%)	74	61	**0.004**
Yes	21 (13.5%)	4	17	
TNM stage				
I + II	126 (80.8%)	67	59	0.154
III + IV	30 (19.2%)	11	19	
Differentiation grade				
I + II	51 (32.7%)	28	23	0.495
III	105 (67.3%)	50	55	
Treatment				
Hepatectomy	120 (76.9%)	62	58	0.569
Hepatectomy + TACE	36 (23.1%)	16	20	

*HBsAg* hepatitis B virus surface antigen, *AFP* alpha-fetoprotein, *PVTT* portal vein tumor thrombosis, *TNM* tumor–nodes–metastases, *TACE* transcatheter arterial chemoembolization

Statistically significant P values (P<0.05) were bold processed

### MTP18 knockdown suppresses HCC cell growth by inhibiting G1–S cell cycle transition and inducing cell apoptosis

**Fig. 2:**
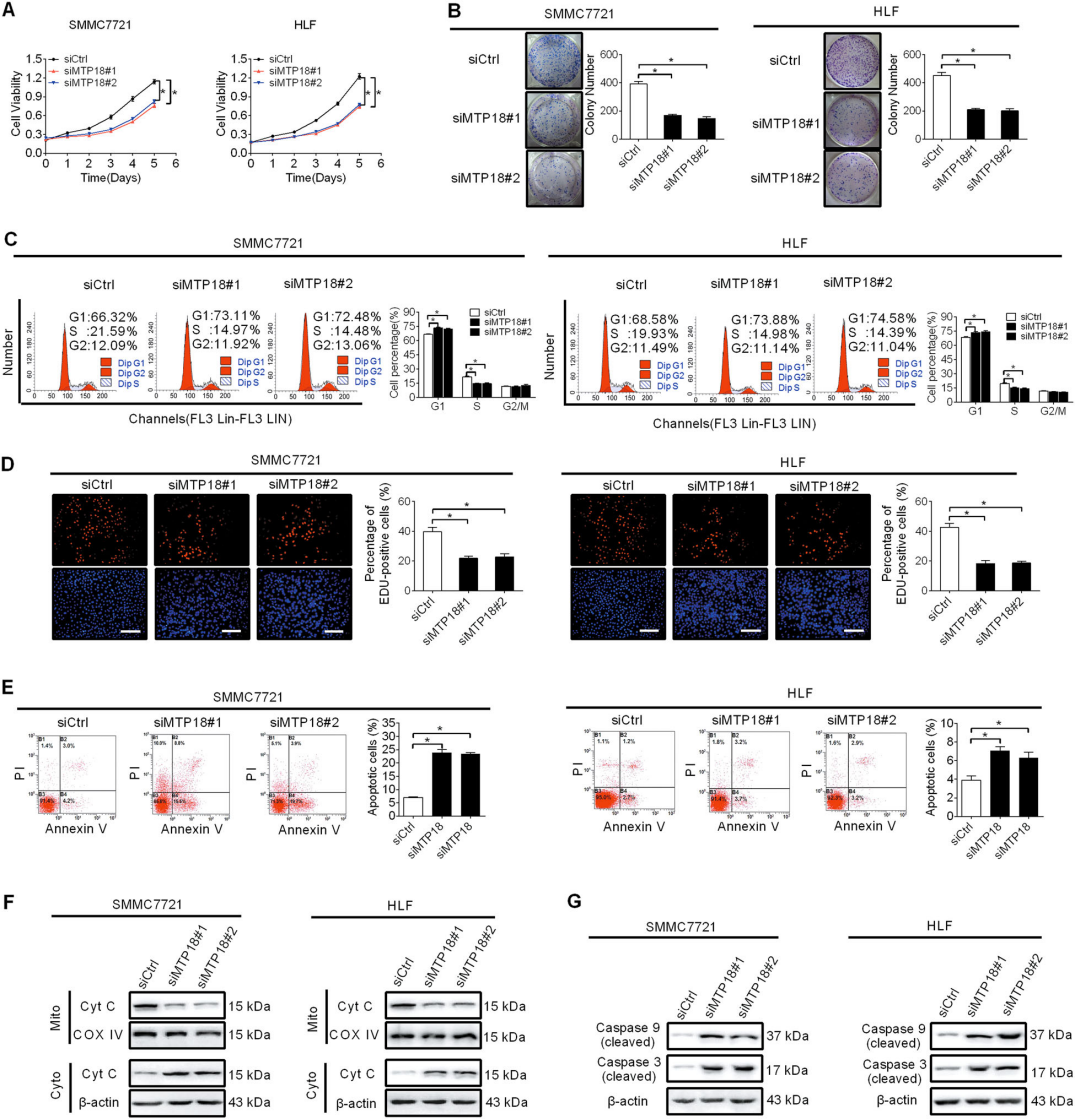
MTP18 knockdown suppresses HCC cell growth by inhibiting G1–S cell cycle transition and inducing cell apoptosis. **a** MTS cell viability analysis in SMMC7721 and HLF cells after transfection with siMTP18 or siCtrl as indicated (siMTP18 siRNA against MTP18, siCtrl control siRNA). **b** Colony formation assay in SMMC7721 and HLF cells with treatment as indicated. **c** Flow cytometry analysis of cell cycle in SMMC7721 and HLF cells with treatment as indicated. **d** EdU incorporation assay for cell proliferation ability was performed in SMMC7721 and HLF cells with treatment as indicated. Scale bars, 50 μm. **e** Flow cytometry analysis of cell apoptosis by Annexin V/ PI staining in SMMC7721 and HLF cells with treatment as indicated. **f** Western blot analyses for protein levels of cytochrome c (cyt c) in cytoplasm and mitochondria of SMMC7721 and HLF cells with treatment as indicated. β-actin and COXIV were used as loading controls for cytoplasm and mitochondria, respectively. Cyto cytoplasm, Mito mitochondria. **g** Western blot analyses for protein levels of cleaved caspase 9 and cleaved caspase 3 in SMMC7721 and HLF cells with treatment as indicated. Data were presented as the mean ± SEM from three independent repeats, *P < 0.05

To elucidate the potential tumor-promoting function of MTP18 in HCC, MTP18 was knocked down by RNA interference in SMMC7721 and HLF cells which have relative high MTP18 expression (shown in Fig. 1c, d). Knockdown of MTP18 was evidenced by qRT-PCR and western blot analysis as shown in Figs. S2A and S2B. Knockdown of MTP18 significantly inhibited cell growth, as evidenced by cell viability and colony formation assays in SMMC7721 and HLF cells (Fig. 2a, b). Increased cell proliferation could be caused by accelerated cell cycle progression or decreased apoptosis, or both. To determine the molecular mechanism by which MTP18 promotes cell growth, we investigated the effects of MTP18 on cell cycle distribution and apoptosis using flow cytometry. MTP18 knockdown resulted in a significant increase of cells in G1 phase and a concomitantly significant decrease of cells in S phase in SMMC7721 and HLF cells (Fig. 2c). Consistently, EdU (5-ethynyl-2’-deoxyuridine) incorporation assay showed significantly fewer proliferating cells in SMMC7721 and HLF with MTP18 knocked down compared with control cells (Fig. 2d). To further explore the mechanisms by which MTP18 may induce G1–S cell cycle, the expression levels of key G1 cell cycle regulators (cyclin D1 and CDK4) and key G1 cell cycle inhibitors (p21 and p27) were detected by western blot analysis. As shown in Fig. S2C, the expression levels of cyclin D1 and CDK4 were significantly downregulated after knockdown of MTP18 in SMMC7721 and HLF cells, while p21 and p27 were significantly elevated. Apoptosis analysis by flow cytometry showed significantly higher percentages of apoptotic cells in SMMC7721 and HLF cells with MTP18 knockdown than those in control cells (Fig. 2e). Consistent with the results from flow cytometry, cytochrome c release and the cleavage of caspase 9 and caspase 3 were significantly induced after MTP18 was knocked down in SMMC7721 and HLF cells (Fig. 2f, g). These results collectively indicate that MTP18 play an important role in the promotion of HCC growth through inducing G1–S cell cycle transition and inhibiting cell apoptosis.

### MTP18 knockdown suppresses invasion and migration of HCC cells through inhibition of epithelial–mesenchymal transition (EMT) and downregulation of MMP9

**Fig. 3:**
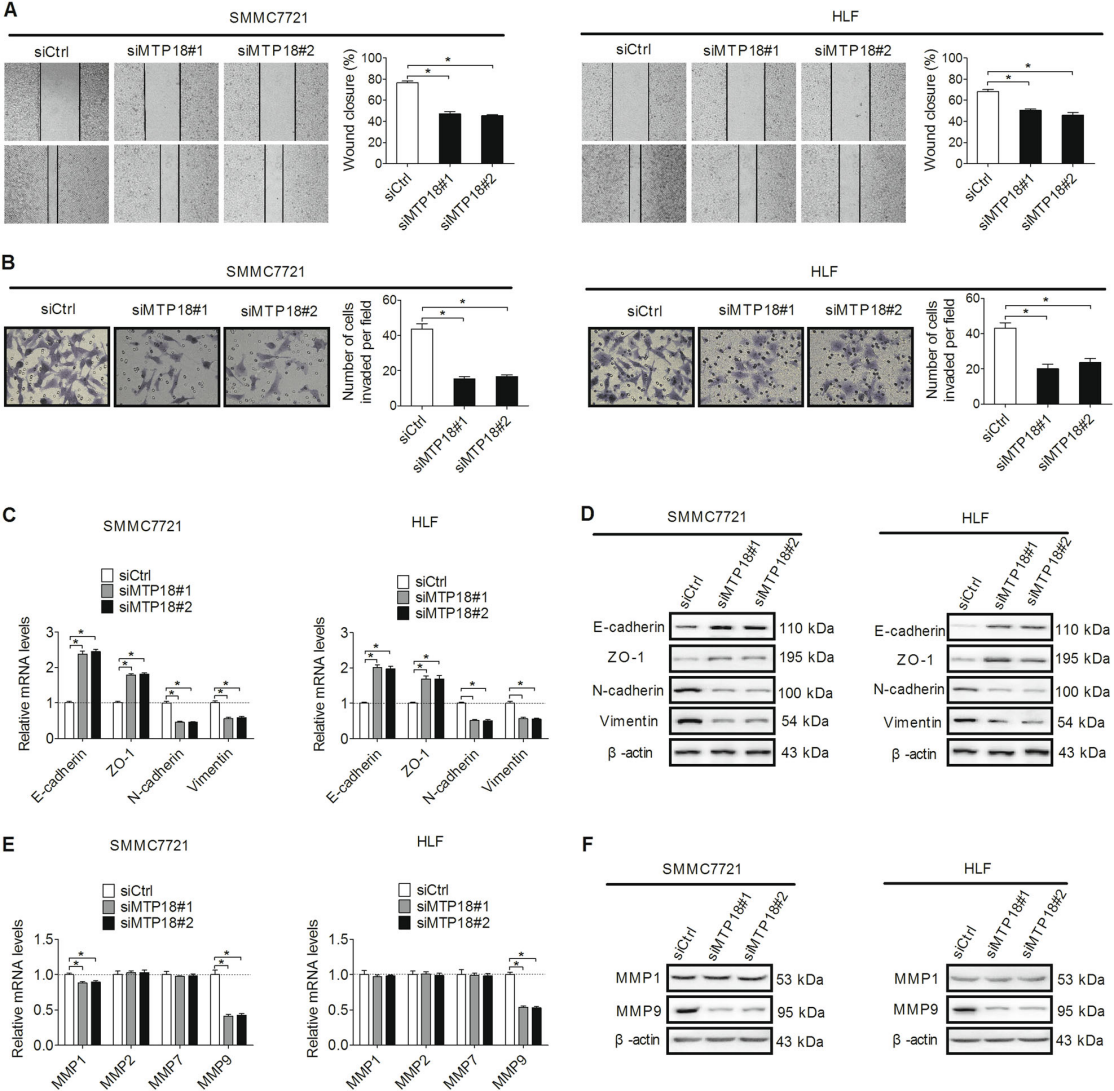
MTP18 knockdown suppresses migration and invasion of HCC cells by inducing epithelial–mesenchymal transition (EMT) and MMP9 production. **a**, **b** Cell migration and invasion abilities were investigated by wound-healing and matrigel invasion assays respectively in SMMC7721 and HLF cells after transfection with siMTP18 or siCtrl as indicated (siMTP18 siRNA against MTP18, siCtrl control siRNA). **c**, **d** Quantitative real-time PCR and western blot analysis for expression levels of EMT markers of E-cadherin, ZO-1, N-cadherin, and Vimentin in SMMC7721 and HLF cells with treatment as indicated. **e** Quantitative real-time PCR analysis for RNA expression levels of MMPs in SMMC7721 and HLF cells with treatment as indicated. **f** Western blot analysis for protein expression levels of MMP-2 and MMP9 in SMMC7721 and HLF cells with treatment as indicated. Data were presented as the mean ± SEM from three independent repeats, *P < 0.05

We also evaluated the effects of MTP18 on the migration and invasion of HCC cells. Wound healing assay showed a significant reduction of wound closure in MTP18 knockdown cells compared with control cells (Fig. 3a). Matrigel invasion assay also indicated that knockdown of MTP18 significantly impaired the invasiveness of SMMC7721 and HLF cells (Fig. 3b).

Epithelial–mesenchymal transition (EMT) plays a critical role in tumor metastasis through reduced cell-cell contact and increased cell motility^14^. We thus clarified whether EMT plays a critical role in MTP18 knockdown-mediated suppression of HCC invasion and migration. qRT-PCR and western blotting analysis showed that MTP18 knockdown significantly increased the expression of epithelial markers (E-cadherin and ZO-1), while reduced the expression of mesenchymal markers (N-cadherin and Vimentin) (Fig. 3c, d). The matrix metalloproteinases (MMPs) also have been widely recognized as major critical molecules assisting tumor cells during metastasis. To further determine whether matrix metalloproteinases (MMPs) play a role in MTP18-mediated HCC cell invasion and metastasis, the expression levels of four matrix metalloproteinases (MMPs), were analyzed by qRT-PCR and western blot analysis in SMMC7721 and HLF cells with MTP18 knockdown. As shown in Fig. 3e, f, only MMP-9 was dramatically downregulated in SMMC7721 and HLF cells with MTP18 knockdown, suggesting that MTP-18 may facilitate the invasion of HCC cells by upregulating MMP9. These findings suggest that MTP18 promotes the migration and invasive abilities of HCC cells both through induction of EMT and upregulation of MMP-9.

### MTP18 knockdown attenuates tumor growth and metastasis in vivo

**Fig. 4:**
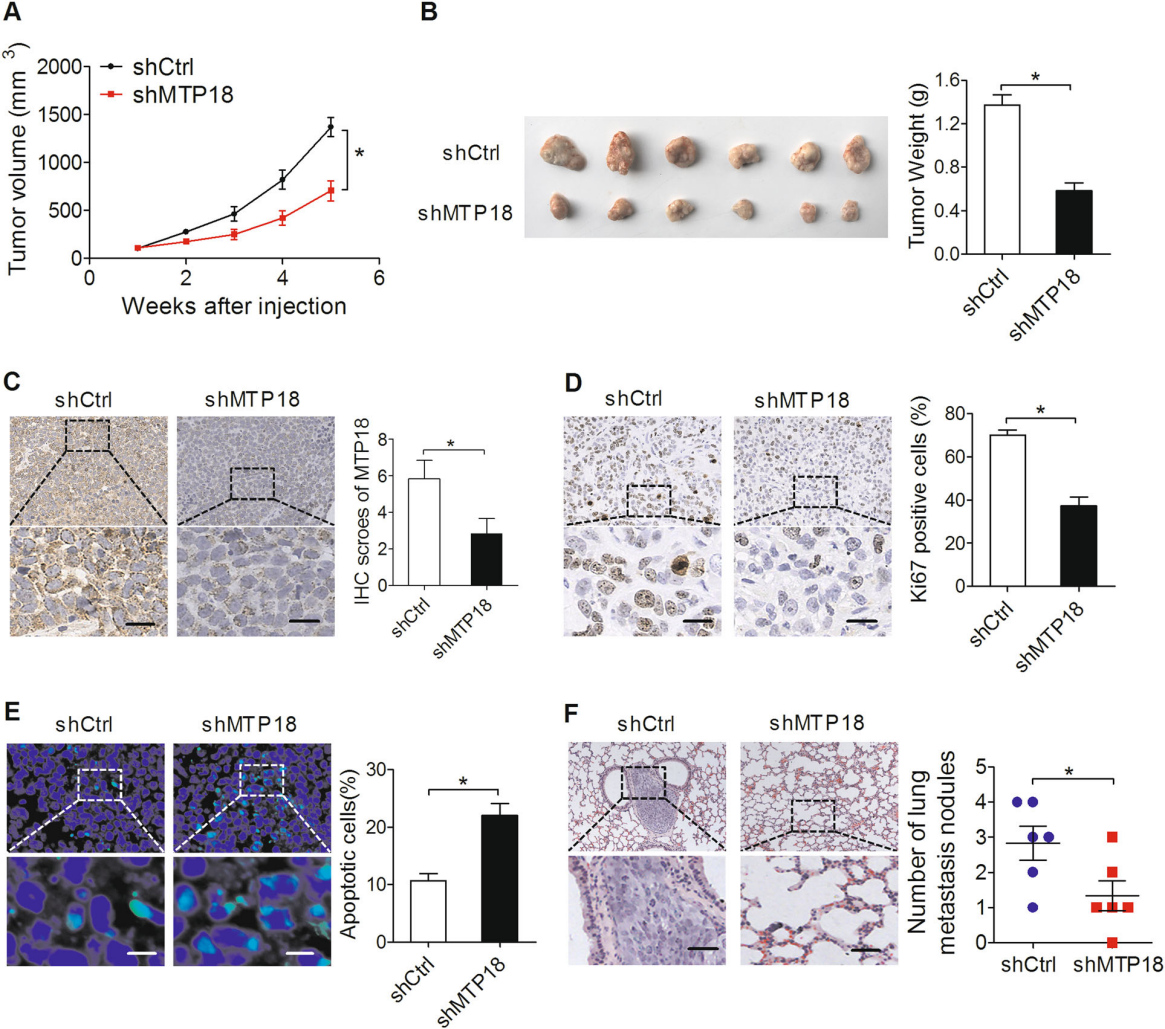
MTP18 knockdown attenuates tumor growth and metastasis in vivo. **a** Tumor growth curves of subcutaneous tumor xenografts established from SMMC7721 cells with stable MTP18 knockdown or control cells. shMTP18 shRNA expression vector against MTP18, shCtrl control shRNA. **b** Tumor tissues (*n* = 6) were dissected from mice at the end of the experiment and the tumor weight was compared between the shMTP18 and shCtrl groups. **c** The expression levels of MTP18 in subcutaneous xenografts were confirmed by immunohistochemistry (IHC). Scale bars, 10 μm. **d** IHC staining for Ki-67 in orthotropic xenografts from each group is shown. **e** TUNEL staining in orthotropic xenografts from each group is shown. Scale bars, 5 μm. **f** The incidences of lung metastases and representative lung metastatic foci in each group are shown. Scale bars, 10 μm. Data were presented as the mean ± SEM from three independent repeats, *P < 0.05

To explore the role of MTP18 in HCC growth in vivo, we established subcutaneous xenograft models by injecting shMTP18 or shCtrl stably transfected SMMC7721 cells in nude mice. Then the status of subcutaneous tumor growth was monitored and compared between the two groups. As shown in Fig. 4a, the shMTP18 group exhibited a much slower tumor growth than those in the shCtrl group. The net wet weight of tumors from shMTP18 group was also significantly lower compared with controls at termination of the experiment (Fig. 4b). Immunohistochemical staining showed significantly decreased expression levels of MTP18 in shMTP18-transfected cells than in the control cells, indicating that the tumor growth suppressive effect was exerted by knockdown of MTP189 (Fig. 4c). In addition, Ki-67 and TUNEL assays showed significantly fewer proliferating cells and more apoptotic cells in shMTP18 xenografts when compared with control (Fig. 4d, e). Moreover, in vivo tail vein cancer metastatic assay further indicated significantly decreased metastatic nodules formed in the lungs in shMTP18 group than those in the shCtrl group (Fig. 4f).

### Overexpression of MTP18 increases growth and metastasis of HCC cells

**Fig. 5:**
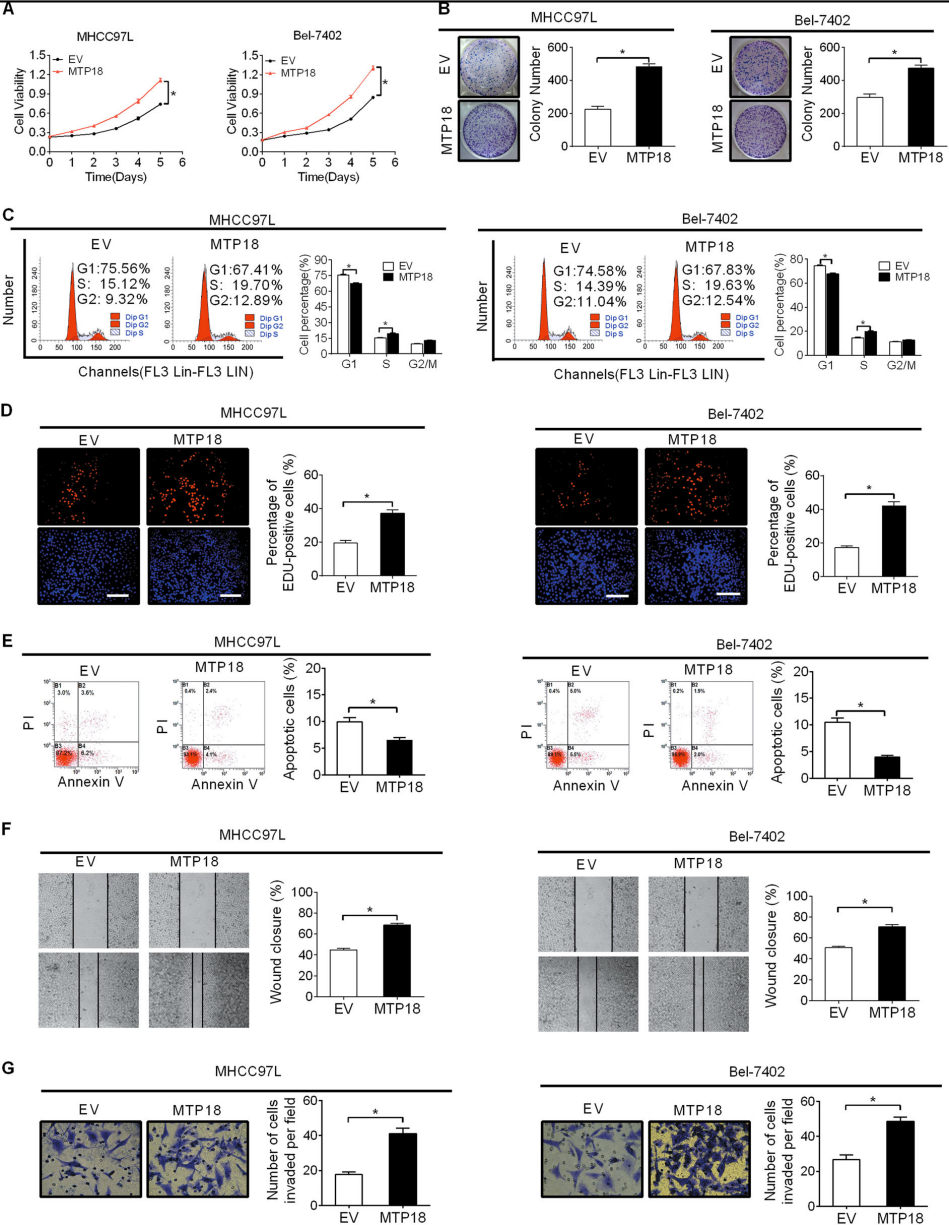
Overexpression of MTP18 increases growth and metastasis of HCC cells. **a**, **b** MTS cell viability and colony formation assays in MHCC97L and Bel-7402 cells after transfection with expression vector encoding MTP18 (MTP18) or empty vector (EV). **c** Flow cytometry analysis for cell cycle distribution in MHCC97L and Bel-7402 cells with treatment as indicated. **d** Cell proliferation ability was evaluated using EdU incorporation assay in MHCC97L and Bel-7402 cells with treatment as indicated. Scale bar, 20 μm. **e** Flow cytometry analysis for apoptosis of MHCC97L and Bel-7402 cells with treatment as indicated. **f**, **g** Wound-healing and matrigel invasion assays in MHCC97L and Bel-7402 cells with treatment as indicated. Data were presented as the mean ± SEM from three independent repeats, *P < 0.05

To further confirm the role of MTP18 in the promotion of HCC cell growth and metastasis, MTP18 was overexpressed by transfecting the MTP18 expression vector or empty vector into MHCC97L and Bel-7402 cell lines, which have relative low MTP18 expression. MTP18 overexpression was evidenced by qRT-PCR and western blot analysis (Fig. S3A and S3B). Forced MTP18 expression in MHCC97L and Bel-7402 cells markedly enhanced cell viability and clonogenicity (Fig. 5a, b). As expected, MTP18 overexpression promoted cell cycle progression and EdU (5-ethynyl-2’-deoxyuridine) incorporation and reduced cell apoptosis (Fig. 5c–e), further supporting the tumor growth-promoting role of MTP18 by causing cell cycle progression and inhibiting apoptosis in HCC. Moreover, MTP18 overexpression significantly increased cell migration and invasion ability both in MHCC97L and Bel-7402 cells, as evidenced by wound-healing and matrigel invasion assays (Fig. 5f, g). Together, these results further confirm a crucial role for MTP18 in the promotion of tumor growth and metastasis.

### Overexpression of MTP18 is mediated by downregulation of miR-125b

**Fig. 6:**
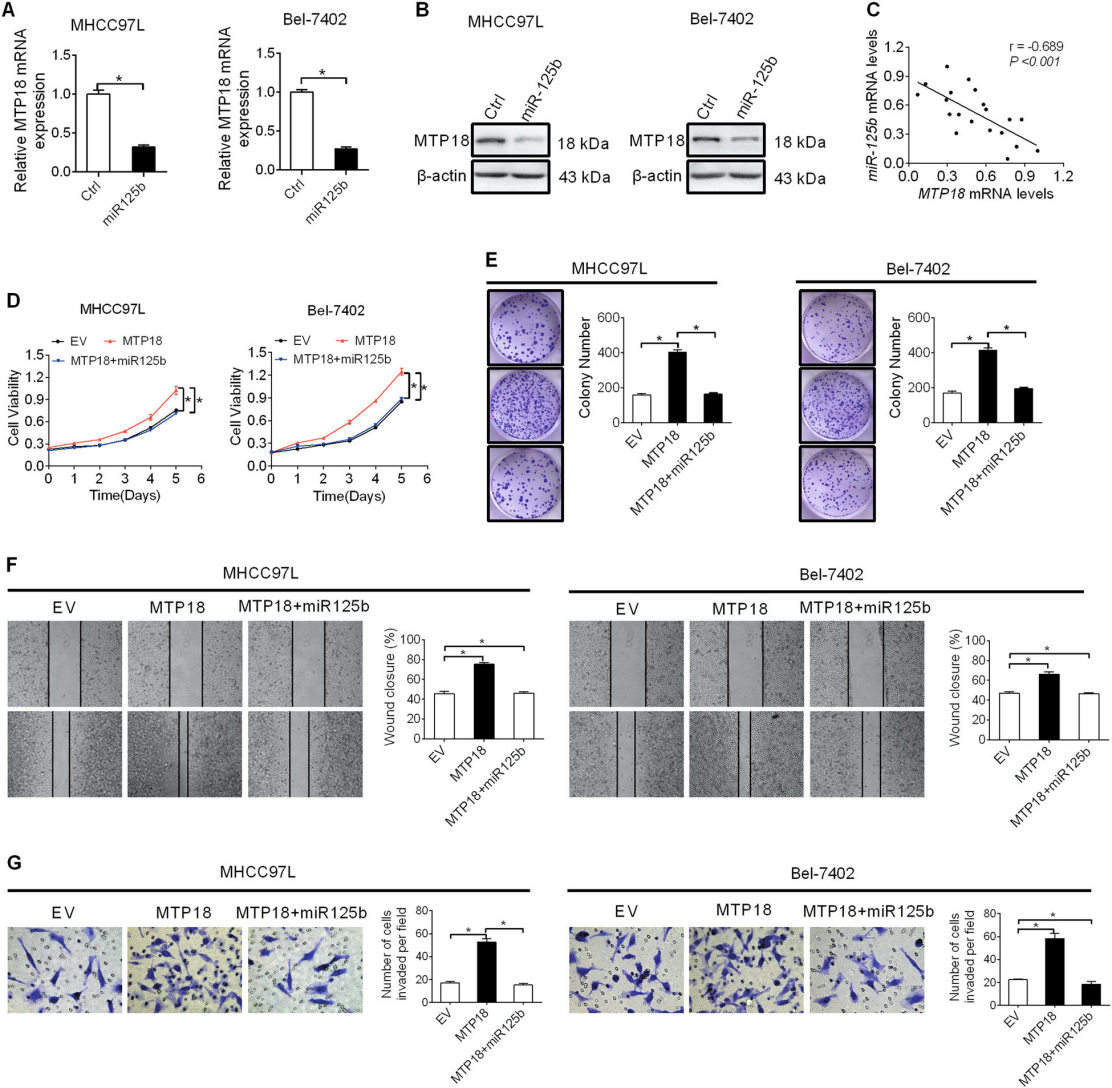
Elevation of MTP18 is mediated by downregulation of miR-125b in HCC cells. **a**, and **b** qRT-PCR and western blot analysis for MTP18 expression in MHCC97L and Bel-7402 cells transfected with the synthetic miR-125b precursor. **c** The relationship between mRNA levels of MTP18 and miR-125b was examined based on the results from qRT-PCR analysis. **d**, **e** MTS cell viability and colony formation assays in MHCC97L and Bel-7402 cells with treatment as indicated. **f**, **g** Wound-healing and matrigel invasion assays in MHCC97L and Bel-7402 cells with treatment as indicated. Data were presented as the mean ± SEM from three independent repeats, *P < 0.05

**Fig. 7:**
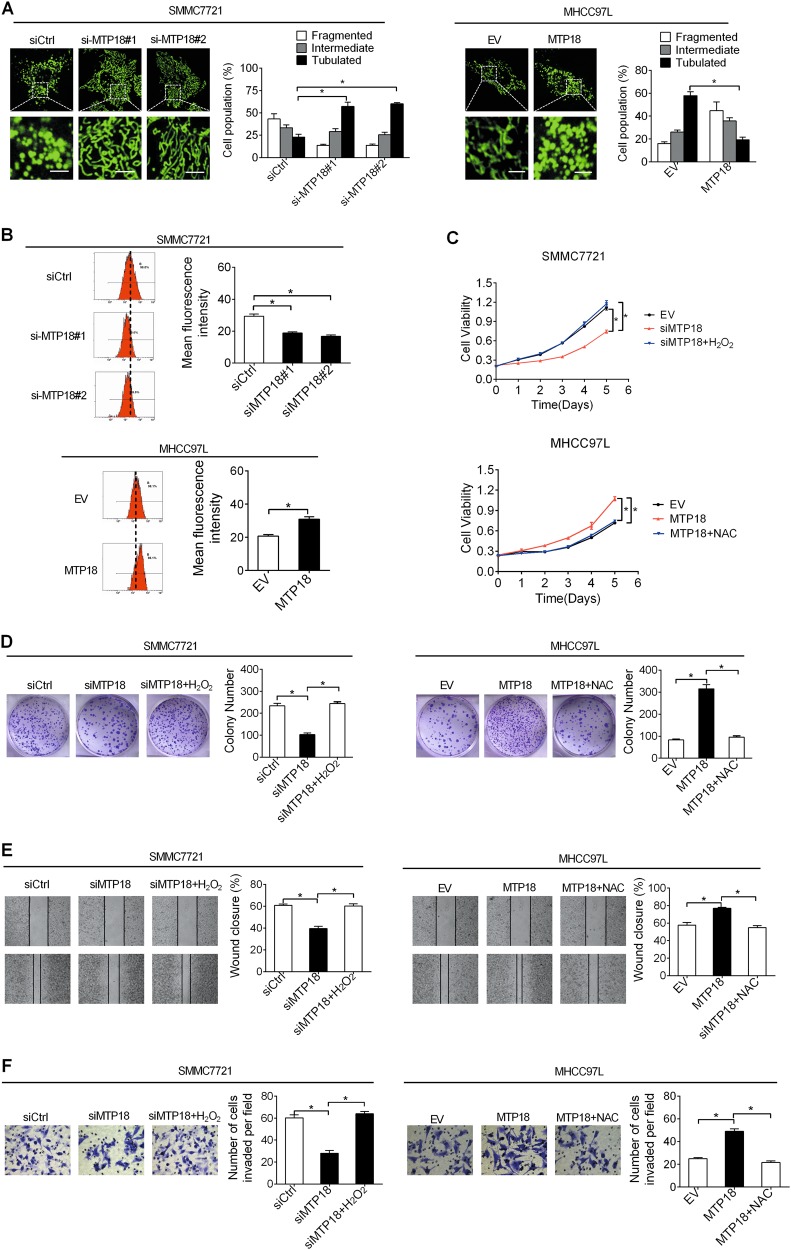
MTP18 promotes tumor growth and metastasis through induction of mitochondrial fission and ROS production. **a** Left panel: Representative confocal microscope images of mitochondrial morphology in SMMC7721 cells with MTP18 knockdown and MHCC97L cells with MTP18 overexpression. Scale bars, 1 μm. Right panel: The proportion of HCC cells (*n* = 50 cells for each sample) with tubulated, intermediate, and fragmented mitochondria was quantified. **b** Detection of intracellular ROS levels by flow cytometry in HCC cells with treatment as indicated. **c**, **d** MTS cell viability colony formation assays in HCC cells treated with 90 μM H_2_O_2_ or 30 mM NAC for 12 h as indicated. **e**, **f** Wound-healing cell migration and transwell invasion analysis in HCC cells treated with 90 μM H_2_O_2_ or 30 mM NAC for 12 h as indicated. Data were presented as the mean ± SEM from three independent repeats, *P < 0.05

MicroRNA is a crucial component in the gene-expression regulation network. MiR-125b has been proved as a frequently downregulated tumor suppressor in HCC. Because a previous study reported that MTP18 is a novel target of miR-125b^15^. We hypothesized that miR-125b should be involved in the upregulation of MTP18 in HCC cells. To test this possibility, synthetic miR-125b precursor was transfected into SMMC7721 and HLF cells. Enforced expression of miR-125b significantly decreased the expression of both mRNA and protein levels of MTP18 in HCC cells, as evidenced both by qRT-PCR and western blot analysis (Fig. 6a, b). In addition, we found a significant negative correlation between the levels of miR-125b and MTP18 in 20 tumor tissue samples from HCC patients (Fig. 7c). Moreover, forced expression of miR-125b significantly reversed both the tumor growth and metastasis-promoting effects of MTP18 in HCC cells (Fig. 6d–g).

### MTP18 promotes tumor growth and metastasis through induction of mitochondrial fission and ROS production

To explore the molecular mechanistic basis of the tumor-promoting effect of MTP18 in HCC, we first assessed the morphological changes of mitochondria in HCC cells with different levels of MTP18. Confocal microscopy of MitoTracker Green staining revealed extensive elongation of mitochondrial after knockdown of MTP18 in SMMC7721 cells, while overexpression of MTP18 resulted in a remarkable fragmentation of mitochondria in MHCC97L cells (Fig. 7a). To explore whether other mitochondrial fission factors have similar functions as MTP18 in HCC, FIS1 (mitochondrial fission protein 1), another novel molecule that is involved in mitochondrial fission, was knocked down by RNA interference in SMMC7721 cells (Fig. S4A). Cell viability, colony formation, wound closure, and matrigel invasion assays showed that knockdown of FIS1 by RNA interference in SMMC7721 remarkably inhibited both cell growth (Fig. S4B and S4C) and metastasis (Fig. S4D and S4E) abilities of HCC cells, suggesting that MTP18 may exert its oncogenic function by regulating the mitochondrial morphology.

Mitochondria are well known as a major source of reactive oxygen species (ROS), which play a central role in the regulation of tumor growth and metastasis by regulating several key oncogenic signaling pathways^16^. Therefore, we hypothesized that mitochondrial fission-mediated ROS production may be involved in the promotion of tumor growth and metastasis by MTP18 in HCC. Flow cytometry analysis indicated that knockdown of MTP18 significantly reduced the production of ROS, while MTP18 overexpression significantly elevated the ROS levels (Fig. 7b). To test whether the oncogenic phenotype induced by MTP18 is associated with elevated production of ROS, we treated HCC cells with H_2_O_2_ or NAC (a ROS scavenger)^17^ to change their ROS levels. Our results show that H_2_O_2_ treatment significantly promoted the growth and metastasis of SMMC7721 cells suppressed by MTP18 knockdown, as evidenced by cell viability, colony formation, wound-healing, and transwell invasion assays. In contrast, NAC treatment could block the growth and metastasis of MHCC97L cells induced by MTP18 overexpression, as evidenced by wound-healing and transwell invasion assays (Fig. 7c–f), indicating that increased mitochondrial fission and subsequent ROS production was involved in the oncogenic property of MTP18 in HCC.

Except for increased ROS production, several previous studies also have linked mitochondrial fission to the aerobic glycolysis^18–20^, also known as Warburg effect, which is considered to be the root of cancer development and progression^21^. We thus analyzed the changes of glycolysis and mitochondrial oxidation when MTP18 was knocked down. Results showed that SMMC7721 cells with MTP18 knockdown exhibited a much lower glucose uptake (Fig. S5A) and lactate production (Fig. S5B) but higher oxygen consumption rate (Fig. S5C) when compared to the control cells. Similar metabolic changes were also observed in SMMC7721 cells after FIS1 was knocked down (Fig. S5A-S5C). These results indicate that mitochondrial fission-mediated aerobic glycolysis may also be involved in the oncogenic property of MTP18 in HCC.

## Discussion

In the present study, we have shown that MTP18 is commonly upregulated in hepatocellular carcinoma (HCC) cell lines and primary HCC tissues due to the downregulation of miR-125b. Kaplan–Meier survival curve analysis revealed that HCC patients with high expression levels of MTP18 had significantly poorer OS and RFS than those with low MTP18 expression levels. A previous study also has shown that DRP1, another regulator of mitochondrial fission, was remarkably upregulated in HCC cells and its upregulation is closely associated with poor prognosis in HCC patients. These observations support the interpretation that mitochondrial hyper-fission is a critical factor for tumor progression.

MiR-125b is a highly conserved miRNA that has multiple targets, and its dysregulation has been reported in many different types of cancer, especially in HCC^22^. MiR-125b has been well established as a frequently downregulated tumor suppressor in HCC, which is involved in almost all the process of HCC, including cell apoptosis, proliferation, migration, and invasion^23–28^. Our present study showed that miR-125b was involved in the overexpression of MTP18 in HCC, suggesting that miR-125b is a critical repressor of MTP18 expression. However, we still cannot rule out the possibility that other regulators may be involved in the upregulation of MTP18 in HCC, such as miR-652-3p, which has also been identified as a direct regulator of MTP18. Therefore, the possibility of involvement of other regulators in MTP18 overexpression still needs further confirmation.

Upregulation of MTP18 in HCC suggested that MTP18 could function as a potential oncogene in HCC. We therefore investigated the biological functions of MTP18 by gain and loss of MTP18 in human HCC cell lines. Knockdown of MTP18 significantly inhibited cell growth in HCC cell lines SMMC7721 and HLF. Conversely, overexpression of MTP18 significantly enhanced cell growth in HCC cell lines MHCC97L and Bel-7402. Consistently, a previous study also has shown that MTP18 knockdown promoted the proliferation of a human prostate cancer cell line of PC-3 and induced the apoptosis of a human keratinocyte cell line of HaCat^29^. However, the effects of MTP18 on apoptosis among different cancer cell types remain inconclusive. A study in gastric cancer has indicated that MTP18 can also act as a tumor suppressor gene, showing a pro-apoptotic role for MTP18 in chemotherapy-induced gastric cancer cell death. Possible explanations for this difference include different tumor origins and cellular context.

Our results indicated that enhanced mitochondrial network by knockdown of MTP18 or FIS1 suppressed the growth and metastasis of HCC cells, while mitochondrial fragmentation induced by MTP18 overexpression promoted those aggressive phenotypes. Consistently, a previous study in HCC has shown that increased mitochondrial fragmentation by overexpression of DRP1 or knockdown of MFN1 promoted the growth of HCC cells both in vitro and in vivo, whereas enhanced mitochondrial network by knockdown of dynamin-related protein 1 (DRP1) or overexpression of mitofusin-1 (MFN1) exhibited an opposite effect^12^. In addition, in several other types of cancer including colon, lung, and breast cancer cells, it also has been shown that enhanced mitochondrial network mediated by inhibition of mitochondrial fission significantly suppress the growth of tumor cells through promotion of cell cycle arrest or induction of cell apoptosis^10,30,31^. Moreover, similar results were also obtained in cardiomyocyte, showing that enhanced mitochondrial network could significantly induce cell apoptosis^32,33^. Collectively, all these results indicate that dysregulated mitochondrial dynamics plays a critical role in the promotion of tumor growth and metastasis, at least in HCC.

The mechanism by which MTP18 promoted HCC cell growth was mediated by inducing G1–S cell cycle transition and inhibiting cell apoptosis. The mechanisms by which MTP18 induce G1–S cell cycle may due to the activation of cyclin D1 and CDK4, and inhibition of p21 and p27, all of which has been well known as key G1–S cell cycle transition regulators. The significance of MTP18 overexpression on HCC cell migration and invasion was also evaluated. Knockdown of MTP18 in SMMC7721 and HLF cells significantly inhibited their migration and invasion abilities, while overexpression of MTP18 in MHCC97L and Bel-7402 showed the opposite effects. The molecular mechanism by which MTP18 exerts its pro-invasive role was shown to be mediated by promotion of (EMT) and upregulation of MMP9. MTP18, also known as MTFP1 is localized in the mitochondria inner membrane and involved in the promotion of mitochondrial fission. Increased mitochondria fission has been shown play a promoting effect on the production of intracellular ROS, which is critical for tumor progression^12,34^. Our study showed a similar effect of MTP18-mediated mitochondrial fission on the production of ROS in HCC cells. Moreover, we found that elevated ROS was involved in the promotion of HCC growth and metastasis by MTP18. Except for increased ROS production, we also demonstrated that MTP18 significantly contributed to the aerobic glycolysis, a phenomenon considered to be the root of cancer development and progression^21^, of HCC cells. This suggests that increased glycolysis may also be involved in the oncogenic property of MTP18 in HCC, which still needs further investigation.

**Fig. 8:**
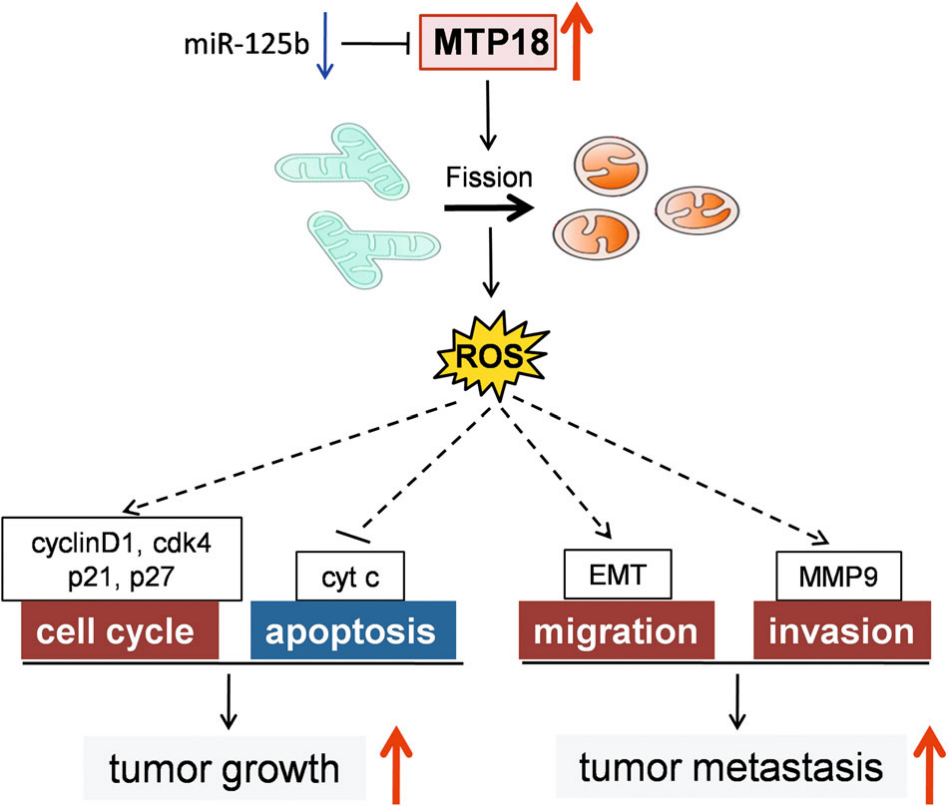
Schematic depicting the effect of MTP18 overexpression on tumor growth and metastasis of HCC and their underlying mechanisms. MTP18 is commonly overexpressed in HCC tissues due to the downregulation of miR-125b, which significantly contributed to the poor prognosis of HCC patients. MTP18 promotes both the growth and metastasis of HCC cells by inducing the progression of cell cycle, epithelial to mesenchymal transition (EMT) and production of MMP9 and suppressing cell apoptosis. Increased mitochondrial fission and subsequent ROS production are involved in the promotion of growth and metastasis by MTP18 in HCC cells

In summary, our date indicate for the first time that MTP18 is frequently overexpressed in HCC and overexpression of MTP18 plays a pivotal oncogenic role in hepatocellular carcinogenesis by promotion of both tumor growth and metastasis of HCC (Fig. 8). Our study suggests that MTP18 may serve as a novel prognostic factor and a therapeutic target in HCC.

## Materials and methods

### HCC cell lines and tissues sample collection

All human HCC cell lines including MHCC97L, SMMC7721, Bel-7402, HepG2, HLF, HCCLM3, and Huh-7, as well as normal human hepatocyte HL-7702, were obtained from the Shanghai Cell Bank of the Chinese Academy of Sciences, Shanghai, China. Cells were cultured in Dulbecco’s Modified Eagle’s medium (DMEM) or RPMI-1640 medium supplemented with 10% fetal bovine serum (Hyclone). In addition, 156 primary hepatocellular carcinoma (HCC) tumor tissue samples were collected during surgical resection at the First Affiliated Hospital of Xi’an Jiaotong University in Xi’an, China. All samples were histologically confirmed as hepatocellular–carcinoma and all subjects provided informed consent for obtaining the study specimens.

### Knockdown and forced expression of target genes

Cells were transfected with expression vectors for MTP18 or siRNA against MTP18 or FIS1 using lipofectamine 2000 (Life Technologies) according to the manufacturer’s instructions, respectively. In addition, synthetic miR-125b precursor (pre-miR-125b; Ambion) and miR-125b antagonist (anti-miR-125b; Ambion, Darmstadt, Germany) were used to change the function of miR-125b. MTP18 and FIS1 RNA interference sequence were 5′-AGAAGGCAGGAGAGGTGCCAAGCCCTGAA-3′ (siMTP18#1), 5′-CCATTGACAGGTCGGTAGACTTCCTCCTG-3′ (siMTP18#2), 5′-AACGAGCUGGUGUCUGUGGAG-3′ (siFIS1#1), and 5′-CCGGCUCAAGGAAUACGAGAA-3′ (siFIS1#2). A scramble form was used as a control: 5′-GCACTACCAGAGCTAACTCAGATAGTACT-3′. The pSilencer™ 3.1-H1 puro vector (Ambion) was used for generation of shRNA expression vectors targeting MTP18. Transfections were performed using lipofectamine 2000 (Invitrogen) according to the manufacturer's protocols. For MTP18 overexpression, the coding sequences of MTP18 were amplified from cDNA derived from Bel-7402 cells and cloned into the pcDNA^TM^3.1(C) vector (Invitrogen, V790-20).

### RNA extraction and real-time PCR analyses

Total RNA was extracted from HCC cell lines or tissue samples using Trizol reagent (Invitrogen) and then reverse transcribed into cDNA with random primers. Real-time PCR was performed on a Corbett 6200 using SYBR Green master mixture (Applied Biosystems, Foster City, CA). All primer sequences used are listed in supplementary Table 2.

### Western blot analysis

Cells or tissues were lysed in RIPA buffer (1%SDS) and protein concentration was determined by the BCA assay (Bio-Rad, Hercules, CA). Equal amount of protein were electrophoresed on SDS–polyacrylamide gels and transferred to polyvinylidene fluoride (PVDF) membranes. After blocked with 5% milk in TBST, membranes were incubated with specific primary antibody and secondary antibody, respectively. Finally, the reactions were detected by enhanced chemiluminescence system (ECL; Amersham Pharmacia Biotech). All primary antibodies used and their working concentrations are listed in supplementary Table 3.

### Immunohistochemistry analysis

Immunohistochemistry was performed using a IHC detection kit (Invitrogen) according to the manufacturer’s instructions. Briefly, paraffin-embedded tissue sections were deparaffinized, rehydrated and blocked for endogenous peroxidase, followed by treatment with hot citrate buffer (pH = 6) under pressure to unmask epitopes. Then the sections were incubated with primary antibody against MTP18 (Proteintech, Wuhan, China) followed by detection using the IHC kit (Invitrogen), and counter-staining with hematoxylin. Finally, MTP18 staining was scored by assigning the percentage of positive cells and their staining intensity.

### Cell viability assay

Cell viability was monitored by determined by the MTS assay (Promega, G3581) in at least triplicate samples according to the manufacturer’s protocol. Briefly, HCC cells were counted and plated onto 96-well cell culture plates at a density of 1000 cells/well. After grown overnight, 20 ml of MTS (0.2%)-PMS (0.092%; phenazine methosulfate, 20:1) solution was added to the 96-well plates and incubation incubated for 2 h. Finally, cell viability was measured by absorbance of 490 nm in a spectrophotometer at 0, 1, 2, 3, 4 and 5 days after transfection.

### Colony formation assay

HCC cells were counted and seeded to 6-well plates (1000 cells). Then, cells were cultured in a 37 °C incubator for approximately 14 days. After that, colonies were fixed and stained with crystal violet, and the colonies were pictured and counted. The assay was carried out in triplicate wells for three independent experiments.

### Apoptosis assays

Cell apoptosis was determined by flow cytometry after staining with Annexin V (FITC-conjugated) apoptosis kit (F-6012, US Everbright Inc) according to the manufacturer’s protocol. Briefly, HCC cells were collected and stained with 500 mL binding buffer and 5 mL Annexin 5-FITC and PI, respectively, and then incubated for 20 min at room temperature in the dark. Cells were analyzed by flow cytometry (Beckman, Fullerton, CA).

For TUNEL assay staining on HCC tissues, an in situ cell death detection kit form Roche (11684795910) was used according to the manufacturer’s protocol. Quantification was performed by calculating the percentage of TUNEL-positive cells by a fluorescence microscope. Results were expressed as the mean number of TUNEL-positive apoptotic HCC cells in each group.

### Cell cycle analysis

Cell cycle was analyzed by the flow cytometry. Briefly, HCC cells were collected and washed twice in cold phosphate buffered saline (PBS), and fixed in 70% cold ethanol at −20 °C for 12 h. Then, cells were resuspended in 1 mL staining solution with 50 U/mL RNase and 50 μg/mL propidium iodide (PI). After incubation for 40 min at 4 °C in the dark, cell cycle was evaluated by flow cytometry (Beckman, Fullerton, CA), and the population of cells in each phase was calculated.

### Mitochondrial morphology analysis

Mitochondrial morphology was visualized by MitoTracker Green FM staining using a confocal microscopy. Briefly, HCC cells were collected and seeded to a confocal dish, and cultured overnight. Subsequently, cells were fixed in 4% paraformaldehyde (PFA; Sigma) for 30 min and incubated with a MitoTracker green FM probe (Molecular Probes, M7514) for 1.5 h. The immunofluorescence images were taken with an Olympus FV 1000 laser-scanning confocal microscope (Olympus Corporation, Tokyo, Japan). The length of mitochondria was measured with the ImageJ software (NIH, Bethesda, MD).

### Wound-healing assay

Cell migration was assessed using the wound-healing assay. Briefly, HCC cells were counted and seeded to 6-well plates. After grown to 90–95% confluency, a scratch was placed in the middle of the wells with a plastic pipette tip and washed with phosphate buffered saline (PBS). The scratched monolayer of HCC cells were photographed at 0 and 24 h using a light inverted optical microscope.

### Matrigel invasion assays

For matrigel invasion assay, the 24-well transwell chamber was coated with an extracellular matrix on the upper surface (BD companion plate). Then, HCC cells counted and seeded (1 × 10^5^) onto the upper chamber wells and incubated in serum-free medium for 48 h. The penetrated cells were then fixed with 4% PFA and stained with crystal violet and counted.

### Tumor xenograft mouse model

Stable knockdown or overexpression of MTP18 was achieved by stably transfected with shMTP18 or MTP18 expression vector. Then, cells were injected subcutaneously into the dorsal right flank of 4-week-old male Balb/c nude mice (*n* = 6 per group). Tumor volume was measured every 3 days for 5 weeks. After that, the mice were killed and the tumor size and tumor weight were measured. All experimental procedures were approved by the Animal Ethics Committee of the First Affiliated Hospital of Xian Jiaotong University.

### Tail vein metastatic assay

1 × 10^6^ HCC cells with different MTP18 expression levels were injected intravenously through the tail vein into the male Balb/c nude mice (*n* = 6 per group). The mice were housed under standard laboratory conditions and cared for in accordance with the institutional ethical guidelines. The mice were killed 8 weeks after injection and the lungs were dissected and paraffin embedded, followed by staining with hematoxylin and eosin (H&E). Finally the tumor nodules formed in the lungs were counted.

### Aerobic glycolysis assays

Glucose uptake, lactate production and oxygen consumption rate were detected as described in the Supplementary materials and methods.

### Statistical analysis

Experiments were performed independently 3 times, where appropriate. The results are shown as the mean ± SEM. SPSS 17.0 software (SPSS, Chicago, IL) was used for statistical analyses and *P*–value < 0.05 was considered statistically significant. Paired or unpaired Student’s *t*-test was used for comparisons between two groups where appropriate. Correlations between measured variables were tested by Pearson correlation analyses. The OS and RFS were evaluated by the Kaplan–Meier method.

## Electronic supplementary material

supplemental information

## References

[1] Wallace MC, Preen D, Jeffrey GP, Adams LA (2015). The evolving epidemiology of hepatocellular carcinoma: a global perspective. Expert Rev. Gastroenterol. Hepatol..

[2] Ozer Etik D, Suna N, Boyacioglu AS (2017). Management of hepatocellular carcinoma: prevention, surveillance, diagnosis, and staging. Exp. Clin. Transplant..

[3] Gururaja Rao S (2017). Mitochondrial Changes in Cancer. Handbook of experimental pharmacology.

[4] Zong WX, Rabinowitz JD, White E (2016). Mitochondria and Cancer. Mol. Cell.

[5] Hsu CC, Lee HC, Wei YH, Mitochondrial DNA (2013). alterations and mitochondrial dysfunction in the progression of hepatocellular carcinoma. World J. Gastroenterol..

[6] Tondera D (2005). The mitochondrial protein MTP18 contributes to mitochondrial fission in mammalian cells. J. Cell Sci..

[7] Trotta AP, Chipuk JE (2017). Mitochondrial dynamics as regulators of cancer biology. Cell. Mol. life Sci.: CMLS.

[8] Chen H, Chan DC (2017). Mitochondrial Dynamics in Regulating the Unique Phenotypes of Cancer and Stem Cells. Cell Metab..

[9] Zhao J (2013). Mitochondrial dynamics regulates migration and invasion of breast cancer cells. Oncogene.

[10] Rehman J (2012). Inhibition of mitochondrial fission prevents cell cycle progression in lung cancer. FASEB J.: Off. Publ. Fed. Am. Soc. Exp. Biol..

[11] Jin B (2011). Anti-tumour efficacy of mitofusin-2 in urinary bladder carcinoma. Med. Oncol..

[12] Huang Q (2016). Increased mitochondrial fission promotes autophagy and hepatocellular carcinoma cell survival through the ROS-modulated coordinated regulation of the NFKB and TP53 pathways. Autophagy.

[13] Zhan L (2016). Drp1-mediated mitochondrial fission promotes cell proliferation through crosstalk of p53 and NF-kappaB pathways in hepatocellular carcinoma. Oncotarget.

[14] Li L, Li W (2015). Epithelial-mesenchymal transition in human cancer: comprehensive reprogramming of metabolism, epigenetics, and differentiation. Pharmacol. & Ther..

[15] Duroux-Richard I (2016). miR-125b controls monocyte adaptation to inflammation through mitochondrial metabolism and dynamics. Blood.

[16] Idelchik M, Begley U, Begley TJ, Melendez JA (2017). Mitochondrial ROS control of cancer. Semin. Cancer Biol..

[17] Downs I, Liu J, Aw TY, Adegboyega PA, Ajuebor MN (2012). The ROS scavenger, NAC, regulates hepatic Valpha14iNKT cells signaling during Fas mAb-dependent fulminant liver failure. PloS One.

[18] Hagenbuchner J, Kuznetsov AV, Obexer P, Ausserlechner MJ (2013). BIRC5/Survivin enhances aerobic glycolysis and drug resistance by altered regulation of the mitochondrial fusion/fission machinery. Oncogene.

[19] Son MJ (2015). Mitofusins deficiency elicits mitochondrial metabolic reprogramming to pluripotency. Cell death Differ..

[20] Guido C (2012). Mitochondrial fission induces glycolytic reprogramming in cancer-associated myofibroblasts, driving stromal lactate production, and early tumor growth. Oncotarget.

[21] Koppenol WH, Bounds PL, Dang CV (2011). Otto Warburg’s contributions to current concepts of cancer metabolism. Nat. Rev. Cancer.

[22] Shimagaki Tomonari, Yoshizumi Tomoharu, Harimoto Norifumi, Yoshio Sachiyo, Naito Yutaka, Yamamoto Yusuke, Ochiya Takahiro, Yoshida Yoshihiro, Kanto Tatsuya, Maehara Yoshihiko (2017). MicroRNA-125b expression and intrahepatic metastasis are predictors for early recurrence after hepatocellular carcinoma resection. Hepatology Research.

[23] Zhao A (2012). MicroRNA-125b induces cancer cell apoptosis through suppression of Bcl-2 expression. J. Genet. Genomics.

[24] Liang L (2010). MicroRNA-125b suppressesed human liver cancer cell proliferation and metastasis by directly targeting oncogene LIN28B2. Hepatology.

[25] Jia HY (2012). MicroRNA-125b functions as a tumor suppressor in hepatocellular carcinoma cells. Int. J. Mol. Sci..

[26] Tsang FH (2014). Prognostic marker microRNA-125b inhibits tumorigenic properties of hepatocellular carcinoma cells via suppressing tumorigenic molecule eIF5A2. Dig. Dis. Sci..

[27] Kim JK (2013). Sirtuin7 oncogenic potential in human hepatocellular carcinoma and its regulation by the tumor suppressors MiR-125a-5p and MiR-125b. Hepatology.

[28] Pan S (2013). ERManI is a target of miR-125b and promotes transformation phenotypes in hepatocellular carcinoma (HCC). PloS One.

[29] Tondera D (2004). Knockdown of MTP18, a novel phosphatidylinositol 3-kinase-dependent protein, affects mitochondrial morphology and induces apoptosis. J. Biol. Chem..

[30] Inoue-Yamauchi A, Oda H (2012). Depletion of mitochondrial fission factor DRP1 causes increased apoptosis in human colon cancer cells. Biochem. Biophys. Res. Commun..

[31] Qian W (2012). Mitochondrial hyperfusion induced by loss of the fission protein Drp1 causes ATM-dependent G2/M arrest and aneuploidy through DNA replication stress. J. Cell Sci..

[32] Ikeda Y (2015). Endogenous Drp1 mediates mitochondrial autophagy and protects the heart against energy stress. Circ. Res..

[33] Shen T (2007). Mitofusin-2 is a major determinant of oxidative stress-mediated heart muscle cell apoptosis. J. Biol. Chem..

[34] Yu T, Sheu SS, Robotham JL, Yoon Y (2008). Mitochondrial fission mediates high glucose-induced cell death through elevated production of reactive oxygen species. Cardiovasc. Res..

