# Supplementary material for: MTP18 overexpression contributes to tumor growth and metastasis and associates with poor survival in hepatocellular carcinoma
Source: Cell Death Dis. 2018 Sep 20;9(10):956. doi: 10.1038/s41419-018-0987-x (PMC6148245; doi:10.1038/s41419-018-0987-x)
Supplement: Supplementary file 1 — supplemental information [file 41419_2018_987_MOESM1_ESM.docx]

**Supplemental information**

**MTP18 overexpression contributes to tumor growth and metastasis and associates with poor survival in hepatocellular carcinoma**

**Supplemental figures**

**
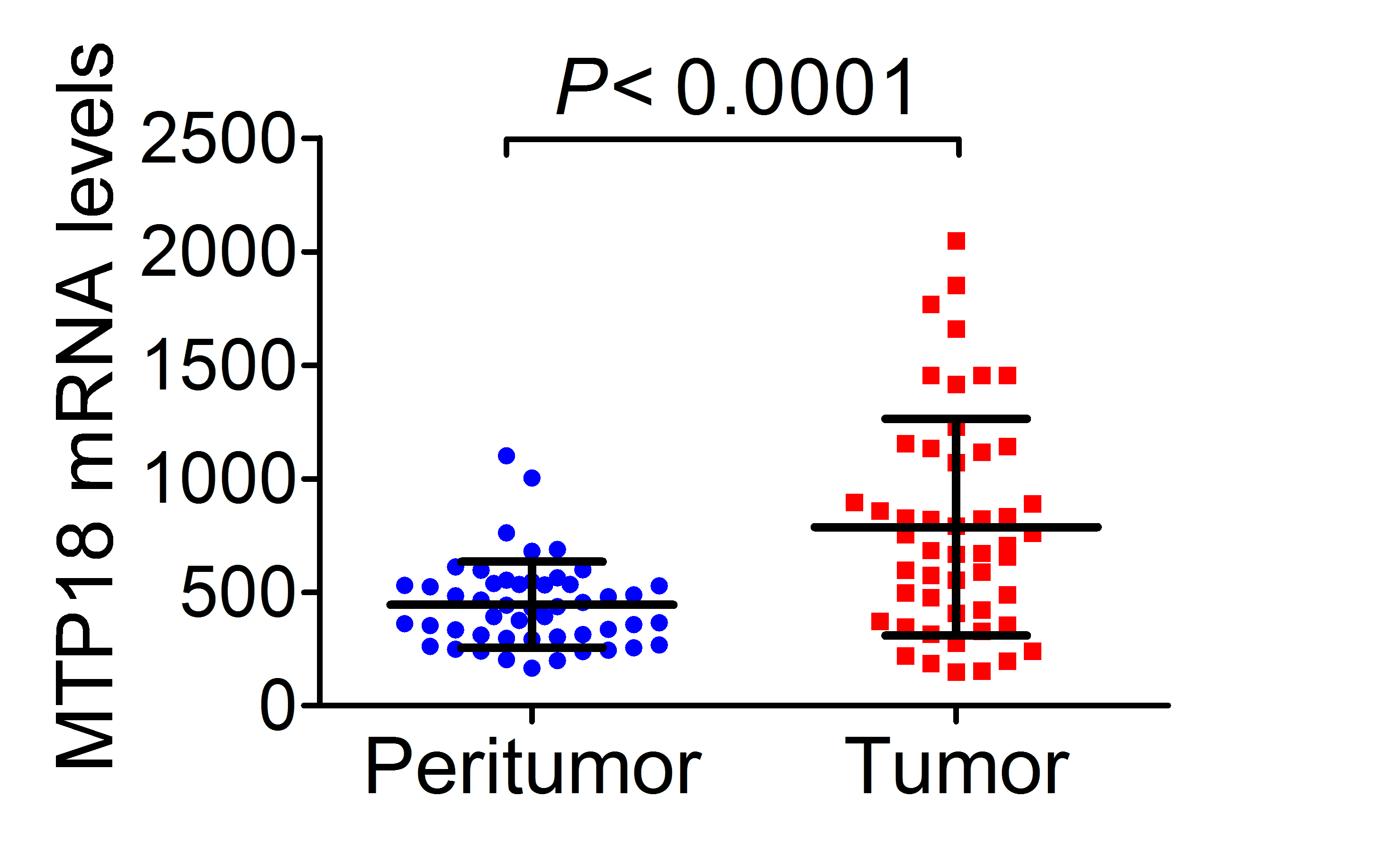
**

**Figure S1. The expression levels of MTP18 in HCC tumor and peritumor were statistically analyzed in the TCGA databases.**

**
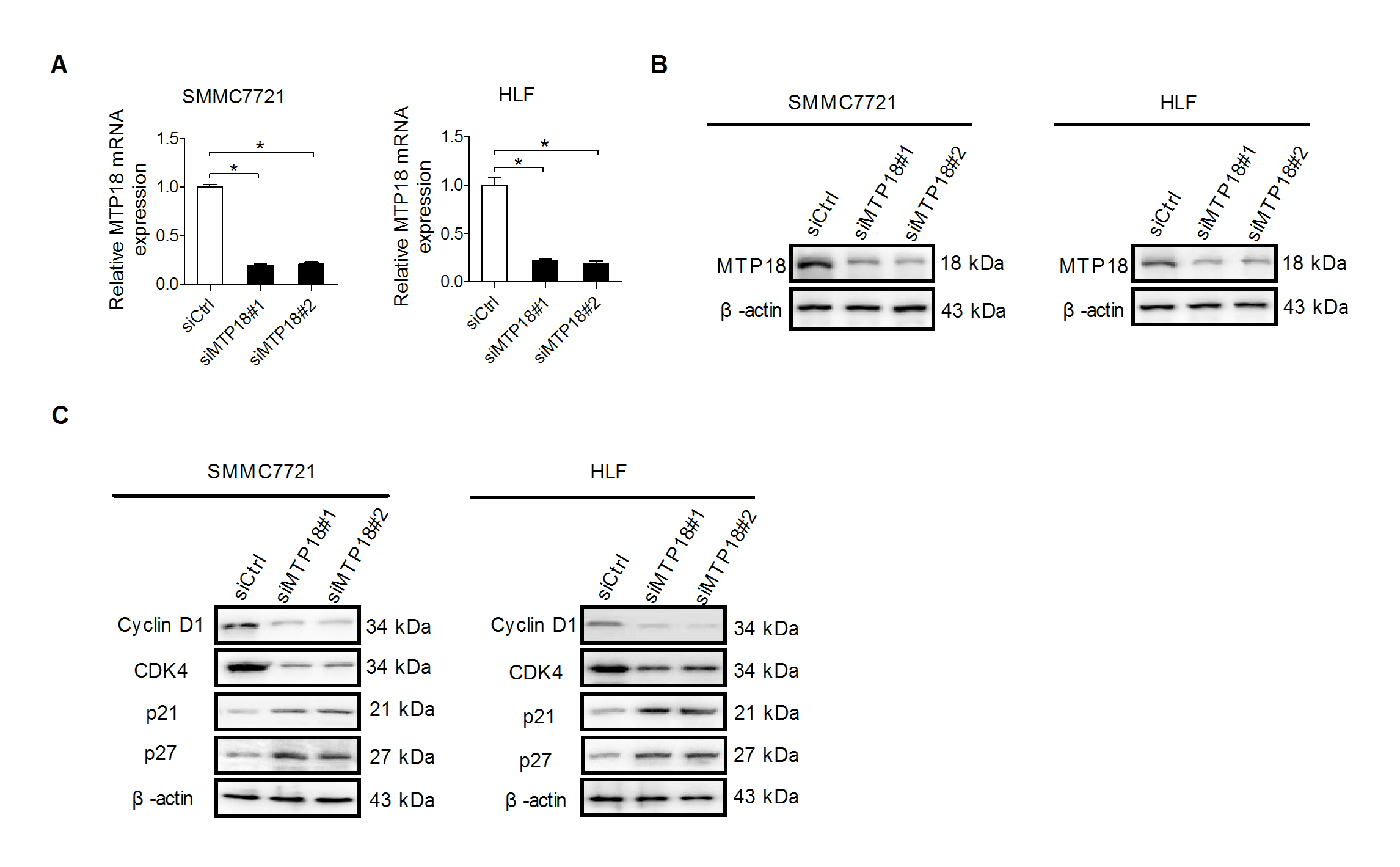
**

**Figure S2. MTP18 knockdown suppressed G1–S cell cycle transition. (A and B)** Knockdown of MTP18 in SMMC7721 and HLF cells was confirmed by quantitative real-time PCR (qRT-PCR) and western blot analysis at mRNA and protein levels. **(C)** Western blot analysis for the protein levels of cell cycle-related protein (cyclin D1, CDK4, p21 and p27) in HCC cells with treatment as indicated. Data were presented as the mean ± SEM from three independent repeats, **P* < 0.05.


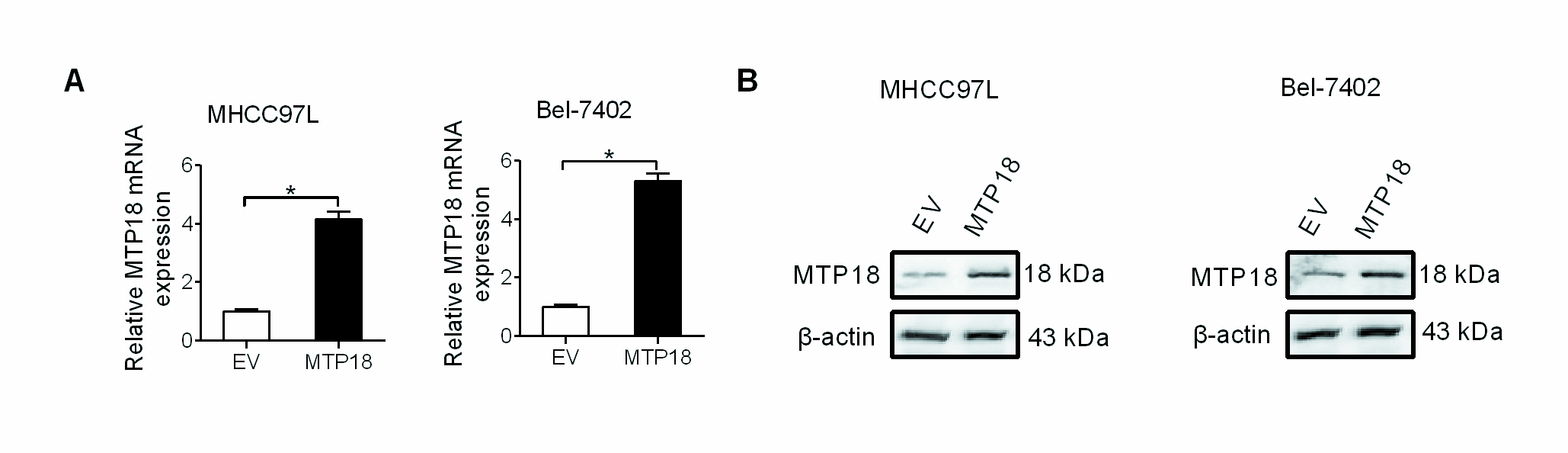


**Figure S3. Forced expression of MTP18 in MHCC97L and Bel-7402 cells.** Overexpression of MTP18 was confirmed by quantitative real-time PCR (qRT-PCR) **(A)** and western blot **(B)** analysis in MHCC97L and Bel-7402 cells after transfection with expression vector encoding MTP18 (MTP18) or empty vector (EV). Data were presented as the mean ± SEM from three independent repeats, **P* < 0.05.

**
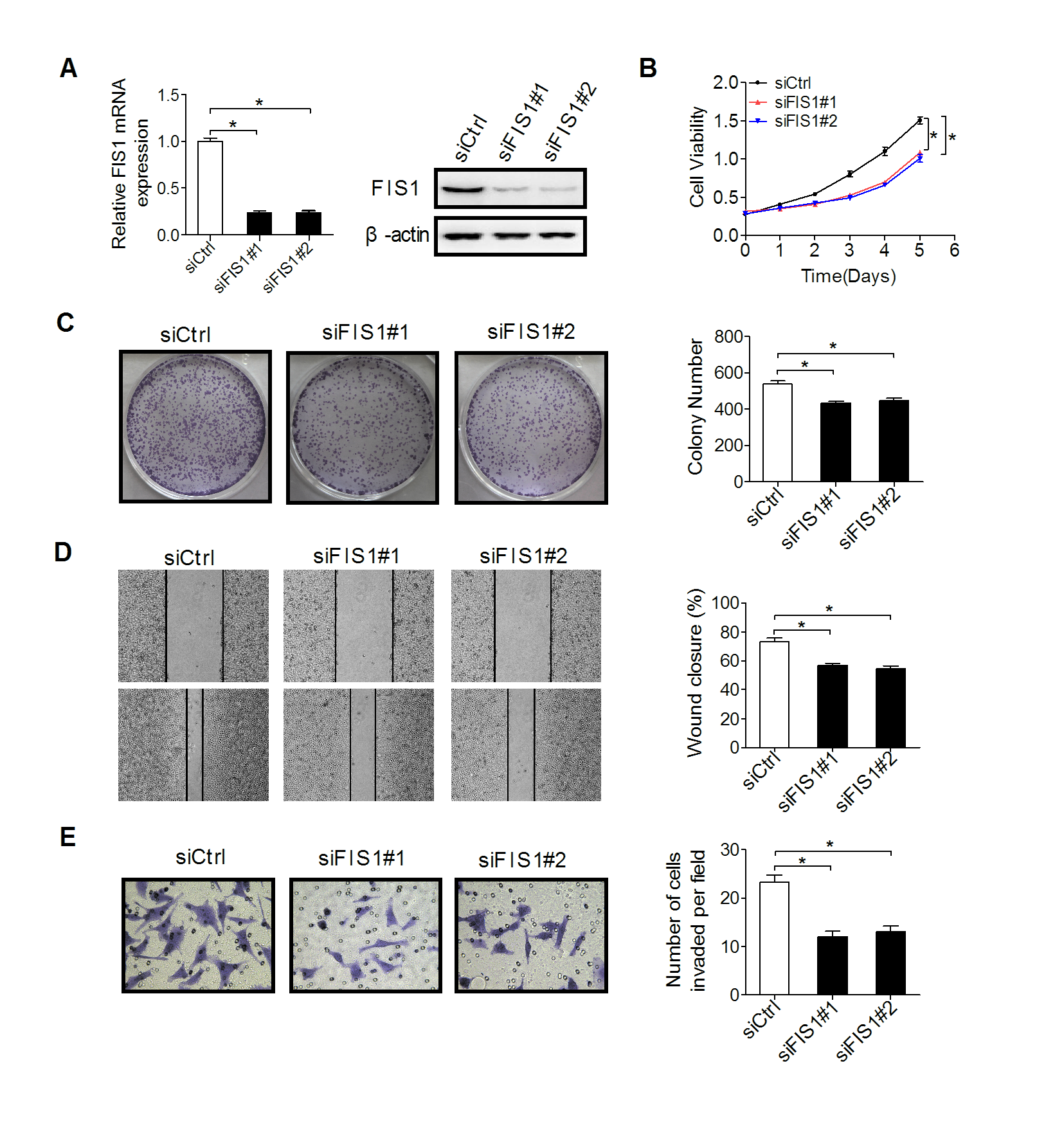
**

**Figure S4. FIS1 knockdown attenuates the in vitro growth and metastasis of SMMC7721 cells. (A)** Knockdown of FIS1 in SMMC7721 cells was confirmed by qRT-PCR and western blot analysis at mRNA and protein levels. **(B and C)** MTS cell viability and colony formation assays in SMMC7721 cells after transfection with siFIS1 or siCtrl as indicated (siFIS1, siRNA against FIS1; siCtrl, control siRNA). **(D and E)** Cell migration and invasion ability were investigated by wound healing and matrigel invasion assays respectively in SMMC7721 cells with treatment as indicated. Data were presented as the mean ± SEM from three independent repeats, **P* < 0.05.

**
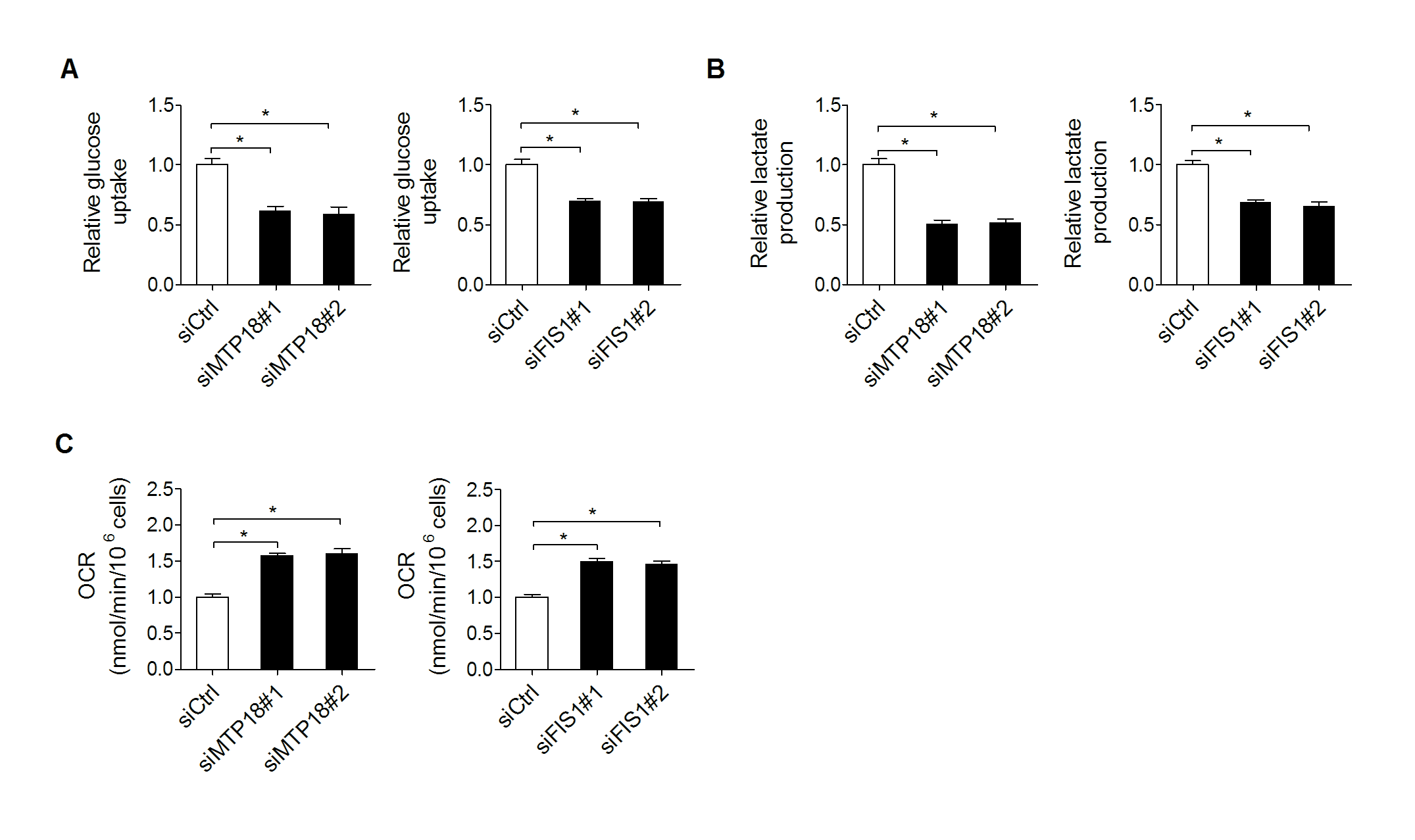
**

**Figure S5. Both MTP18 and FIS1 promoted aerobic glycolysis of HCC cells.** (A) Glucose uptake was measured in SMMC7721 cells after transfection with siMTP18, siFIS1 or siCtrl as indicated. (B) Lactate production were measured in SMMC7721 cells with treatment as indicated. (C) Oxygen consumption rate (OCR) were measured in SMMC7721 cells with treatment as indicated. Data were presented as the mean ± SEM from three independent repeats, **P* < 0.05.

**Supplemental materials and methods**

**Measurement of glucose uptake and lactate production**

Glucose uptake of HCC cells with different levels of MTP18 or FIS1 were determined using the Amplex Re Glucose/Glucose Oxidase Assay kit (Molecular Probes, Carlsbad, CA) according to the manufacturer’s instructions. In addition, lactate productions in the culture medium were detected by using a Lactate Assay Kit (BioVision, Mountain View, CA) following the manufacturer’s protocol. Both results were normalized to the total protein amounts of the detected cells.

**Measurement of oxygen consumption rate**

Cellular O_2_ consumption rate was evaluated using a liquid-phase oxygen electrode system (Hansatech Instruments, Pentney, Norfolk, UK) following the manufacturer’s protocol. The signals from the O_2_ sensor were recorded at intervals of one second.

**Supplementary Tables**

**Table 1. Correlation of MTP18 expression with clinicopathologic features in patients with hepatocellular carcinoma.**

| Variables | No. of cases (%) | MTP18 expression | | *P* value |
| --- | --- | --- | --- | --- |
|  |  | Low | High |  |
| All | 156 (100%) | 78 | 78 |  |
| Age |  |  |  |  |
| <55 | 68 (43.6%) | 32 | 36 | 0.628 |
| >=55 | 88(56.4%) | 46 | 42 |  |
| Gender |  |  |  |  |
| Female | 21 (13.5%) | 13 | 8 | 0.348 |
| Male | 135 (86.5%) | 65 | 70 |  |
| HBsAg |  |  |  |  |
| Negative | 15 (9.6%) | 9 | 6 | 0.588 |
| Positive | 141 (90.4%) | 69 | 72 |  |
| AFP (ug/ml) |  |  |  |  |
| <200 | 85 (54.5%) | 41 | 44 | 0.748 |
| >=200 | 71 (44.5%) | 37 | 34 |  |
| Maximum diameter of lesion |  |  |  |  |
| <5 | 127 (81.4%) | 69 | 58 | **0.038** |
| >=5 | 29 (18.6%) | 9 | 20 |  |
| PVTT |  |  |  |  |
| No | 135 (86.5%) | 74 | 61 | **0.004** |
| Yes | 21 (13.5%) | 4 | 17 |  |
| TNM stage |  |  |  |  |
| I+ II | 126 (80.8%) | 67 | 59 | 0.154 |
| III+ IV | 30 (19.2%) | 11 | 19 |  |
| Differentiation grade |  |  |  |  |
| I+ II | 51 (32.7%) | 28 | 23 | 0.495 |
| III | 105 (67.3%) | 50 | 55 |  |
| Treatment |  |  |  |  |
| Hepatectomy | 120 (76.9%) | 62 | 58 | 0.569 |
| Hepatectomy+ TACE | 36 (23.1%) | 16 | 20 |  |

**Abbreviations**: HBsAg, hepatitis B virus surface antigen; AFP, alpha-fetoprotein; PVTT, portal vein tumor thrombosis; TNM, tumor-nodes-metastases; TACE, transcatheter arterial chemoembolization.

**Table 2.** Sequence of primers for qRT-PCR analysis

| **1. Primers used in q-PCR analysis** | | |  |
| --- | --- | --- | --- |
| *MTP18* | forward primer | TAATCCACCCCATCGACAG | |
|  | reverse primer | TCCACTGACGGGTACAGCTT | |
| *E-cadherin* | forward primer | AAAGGCCCATTTCCTAAAAACCT | |
|  | reverse primer | TGCGTTCTCTATCCAGAGGCT | |
| *Z0-1* | forward primer | CGACCAGATCCTCAGGGTAA | |
|  | reverse primer | TCCATAGGGAGATTCCTTCTCA | |
| *N-cadherin* | forward primer | TCAGGCTGTGGACATAGAAACC | |
|  | reverse primer | GCTGTAAACGACTCTGGCACT | |
| *Vimentin* | forward primer | GACGCCATCAACACCGAGTT | |
|  | reverse primer | CTTTGTCGTTGGTTAGCTGGT | |
| *GAPDH* | forward primer | GGAGCGAGATCCCTCCAAAAT | |
|  | reverse primer | GGCTGTTGTCATACTTCTCATGG | |
| *MMP1* | forward primer | CACAGCTTTCCTCCACTGCTGCT | |
|  | reverse primer | GGCATGGTCCACATCTGCTCTTG | |
| *MMP2* | forward primer | ACCTGGATGCCGTCGTGGAC | |
|  | reverse primer | TGTGGCAGCACCAGGGCA | |
| *MMP7* | forward primer | TGAATTTGGCCACTCTCTGGGTCT | |
|  | reverse primer | TCTGAATGCCTGCAATGTCGTCCT | |
| *MMP9* | forward primer | ACGACATAGACGGCATCCAGTATC | |
|  | reverse primer | AGGTATAGTGGGACACATAGTGGG | |
| *FIS1* | forward primer | GTCCAAGAGCACGCAGTTTG | |
|  | reverse primer | ATGCCTTTACGGATGTCATCATT | |
| *miR-125b* | forward primer | TCCCTGAGACCCTAACTTGTGA | |
|  | reverse primer | Uni-miR qPCR Primer | |

**Table 3.** Primary antibodies used for western blot and immunohistochemistry.

| **Antibody** | **Company (Cat. No.)** | **Working dilutions** |
| --- | --- | --- |
| MTP18 | Proteintech (14257-1-AP) | WB: 1/800 IHC: 1/250 |
| cytochrome c | Proteintech (10993-1-AP) | WB: 1/600 |
| cyclin D1 | Proteintech (60186-1-AP) | WB: 1/8 |
| CDK4 | Proteintech (11026-1-AP) | WB: 1/600 |
| p21 | Proteintech (10355-1-AP) | WB: 1/1000 |
| P27 | Proteintech (25614-1-AP) | WB: 1/3000 |
| COX IV | abcam (ab14744) | WB: 1/750 |
| Caspase 3 | Proteintech (66169-1-Ig) | WB: 1/1000 |
| Caspase 9 | Proteintech (66169-1-Ig) | WB: 1/750 |
| E-cadherin | abcam (ab1416) | WB: 1/1000 |
| Z0-1 | abcam (ab190085) | WB: 1/1000 |
| N-cadherin | abcam (ab98952) | WB: 1/1000 |
| Vimentin | abcam (ab8978) | WB: 1/1000 |
| MMP1 | Proteintech (10371-2-AP) | IHC:1/1000 |
| MMP9 | Proteintech (10375-2-AP) | WB: 1/1000 IHC:1/100 |
| FIS1 | Abcam (ab156865) | WB: 1/1000 |
| β-actin | Proteintech (20536-1-AP) | WB: 1/1000 |
